# Bis-prodrug cryopreserved lipid nanoparticles with enzymatically triggered release

**DOI:** 10.1039/d5na00675a

**Published:** 2026-01-08

**Authors:** Cameron Hogarth, Keith Arnold, Heba Elkateb, Steve Rannard, Tom O. McDonald

**Affiliations:** a Department of Chemistry, University of Liverpool Crown Street Liverpool L69 7ZD UK; b Material Innovation Factory, University of Liverpool Liverpool L7 3NY UK; c Department of Materials, The University of Manchester Oxford Road Manchester M13 9PL UK Thomas.McDonald@Manchester.ac.uk; d Henry Royce Institute, The University of Manchester Oxford Road Manchester UK

## Abstract

Lipid nanoparticle (LNP) formulations have emerged as a versatile platform for the delivery of therapeutics. However, achieving long-term stability and effective delivery of water-soluble small molecule drugs remains a challenge. In this study, we demonstrate a cryopreservable LNP formulation incorporating a hydrophobically modified bis-prodrug of lamivudine. By systematically varying the surfactant composition by combining a PEGylated surfactant (Brij S20) with an unPEGylated, zwitterionic lipid (Lipoid S100), we identify formulations that maintain colloidal stability following freeze–drying and redispersion in the presence of 10% w/v sucrose. Particle size measurements before and after lyophilisation indicate that surfactant ratio significantly impacts redispersibility, with 50/50 Brij/lipoid compositions offering the best performance. A core composition comprising the prodrug and tricaprin at either 1 : 1 or 3 : 1 ratio was evaluated, with the 3 : 1 formulation achieving redispersed particle sizes below 150 nm and low polydispersity. Enzymatic studies using porcine liver esterase confirm slow, sustained conversion of the bis-prodrug to active lamivudine over up to 9 weeks. This work highlights the opportunity of a prodrug-based strategy to formulate water-soluble APIs into stable, freeze-dried LNPs, enabling controlled, enzyme-responsive release. These findings offer insight into how surfactant composition influences freeze–drying compatibility and provide a platform for the development of LNP systems for small molecule delivery.

## Abstract 2

Bis-prodrug-loaded LNPs show robust cryostability and prolonged esterase-mediated drug release after lyophilisation and rehydration.

## Graphical abstract

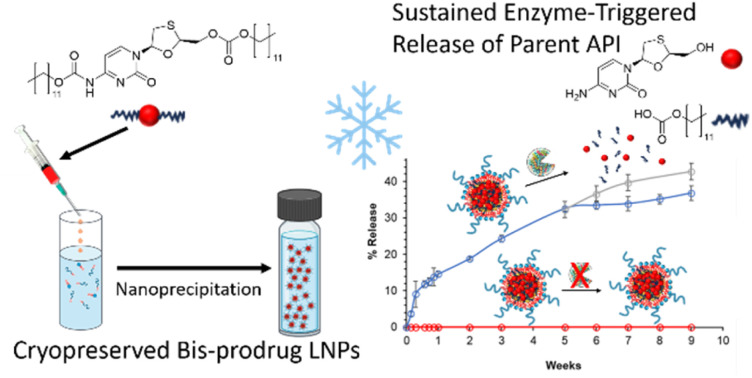

## Introduction

Interest in lipid nanoparticles (LNPs) has expanded greatly since their approval as vaccines for COVID-19, with extensive work undertaken on the delivery of nucleic acids^1^ and particularly mRNA.^2,3^ In addition on the delivery of nucleic acids, LNPs are versatile drug delivery systems that can be used to encapsulate a range of actives including small molecules,^4,5^ biologics^6^ and labels to enable diagnosis.^7,8^ However, there are still a number of challenges associated with the development and wider applications of LNPs. Firstly, LNPs like many other colloidal formulations can suffer from poor long-term stability upon storage.^9,10^ For small molecule drug compounds, stability issues are primarily colloidal (aggregation or growth of the nanoparticles) or due to leakage of the active pharmaceutical ingredients (APIs). To address the limitations of freeze–thaw storage, lyophilisation offers a viable alternative for long-term stabilisation.^11–13^ The freeze–thaw process is a relatively straightforward preservation method, used effectively to maintain the stability of the mRNA COVID-19 Moderna vaccine.^14^ In this approach, formulations are frozen at temperatures as low as −50 °C and thawed prior to use. By freezing, the LNPs are immobilised within an ice matrix, preventing aggregation or leakage until the formulation is thawed and returns to a colloidal state. However, a key limitation of freeze–thaw preservation is the cost associated with maintaining ultra-low temperatures, which restricts accessibility in regions lacking cold chain infrastructure. As an alternative, freeze–drying (lyophilisation) offers a more versatile approach to enhancing LNP stability. Lyophilisation is a multistep process: first, the formulation is frozen, followed by sublimation of ice in a primary drying step, and desorption of residual moisture in a secondary drying step.^15^ This process yields a dry, porous ‘cake’ or ‘scaffold’ in which nanoparticles are embedded, preventing aggregation.^16^ Upon rehydration, the cake can redisperse, restoring the nanoparticles to a colloidal suspension. However, both freeze–thaw and lyophilisation introduce stress to the colloidal system, making the inclusion of stabilising agents, known as cryoprotectants and/or lyoprotectants, essential. Cryoprotectants protect against freezing stresses while lyoprotectants provide protection against drying stresses.^17^ This protection is achieved by providing a protective layer around nanoparticles, increasing viscosity^18^ and potentially forming a glassy matrix at sub-zero temperatures; these behaviours separate particles as ice crystals form, preventing aggregation. Lyoprotectants stabilise particles during and after the sublimation process in freeze–drying. Considerable research has focused on optimising cryoprotectant and lyoprotectant additive materials for lyophilised cakes. For example, Schersch *et al.* have shown that the type and concentration of these additives can determine whether a cake maintains its structural integrity or collapses, affecting redispersibility.^19^ Sylvester *et al.* demonstrated that insufficient additive-to-nanoparticle ratios may lead to structural collapse, resembling cakes without additives.^20^ Common cryoprotectants and lyoprotectants include sugars (*e.g.*, sucrose, trehalose, glucose, and mannitol) and polymers (*e.g.*, polyethylene glycol-based polymers).^21–23^ For example, Trenkenschuh *et al.* showed that sucrose could provide effective stabilisation of LNPs against freeze–thaw stress.^24^ Studies on mRNA loaded LNPs have explored the effects of cryoprotectant type, concentration, buffer conditions, and freezing protocols on colloidal stability, transfection efficiency, and mRNA integrity following freeze–drying and redispersion.^25–28^ These investigations have highlighted the need for careful optimisation of formulation parameters to preserve nanoparticle structure and biological function. However, the role of surfactant composition in conferring cryostability remains underexplored. For LNPs the selection of surfactants in the successful formation of LNPs. Numerous studies have screened different surfactants to identify viable formulations by nanoprecipitation.^29–31^ We have previously shown that combining pegylated and non-pegylated surfactants influences particle size as well as the crystallinity of the lipid.^32^ Surfactants differ in their ability to support the formation of LNPs when different lipids are used.^33^ Changes in the relative hydrophilic/hydrophobic composition of pegylated surfactant can change the internal polarity inside the core of LNPs.^34^ Therefore, it is clear that surfactants provide multi-functional benefits for LNPs, however their influence on stability during freeze thaw or freeze drying needs further study.

An additional challenge for LNPs, is their limited ability to encapsulate water-soluble APIs. One approach to overcome this limitation is to use a pro-drug approach to make the API more hydrophobic. Recently, we have shown that by producing a hydrophobically-modified version of lamivudine, a nucleoside reverse transcription inhibitor using in HIV therapy, it was possible to achieve active loadings of up to 32%.^35^ This hydrophobically modified drug has the potential to behave as a prodrug, whereby cleavage of carbonate and carbamate linkers would release the active.^36^ The potential of this behaviour for LNPs could be highly beneficial by providing the ability for the particles to offer triggered release of active pharmaceutical ingredients in response to enzymes in their surrounding media,^37–39^ or provide long-acting drug delivery.^36^ Many conventional prodrug strategies often focus on single-site hydrophobic modification.^40^ Modification of drug molecules at two sites, often termed bis-produgs, may lead to reduced drug release rate due to lower activation efficiency.^41^ To date, there have been no studies that have shown prodrug activation of lamivudine from LNPs. Lamivudine is a critical component of first-line HIV therapies,^42^ but its water solubility and low molecular weight present challenges for effective encapsulation in LNPs. Addressing these challenges through prodrug strategies offers opportunities for enhanced drug loading and prolonged release.

**Fig. 1:**
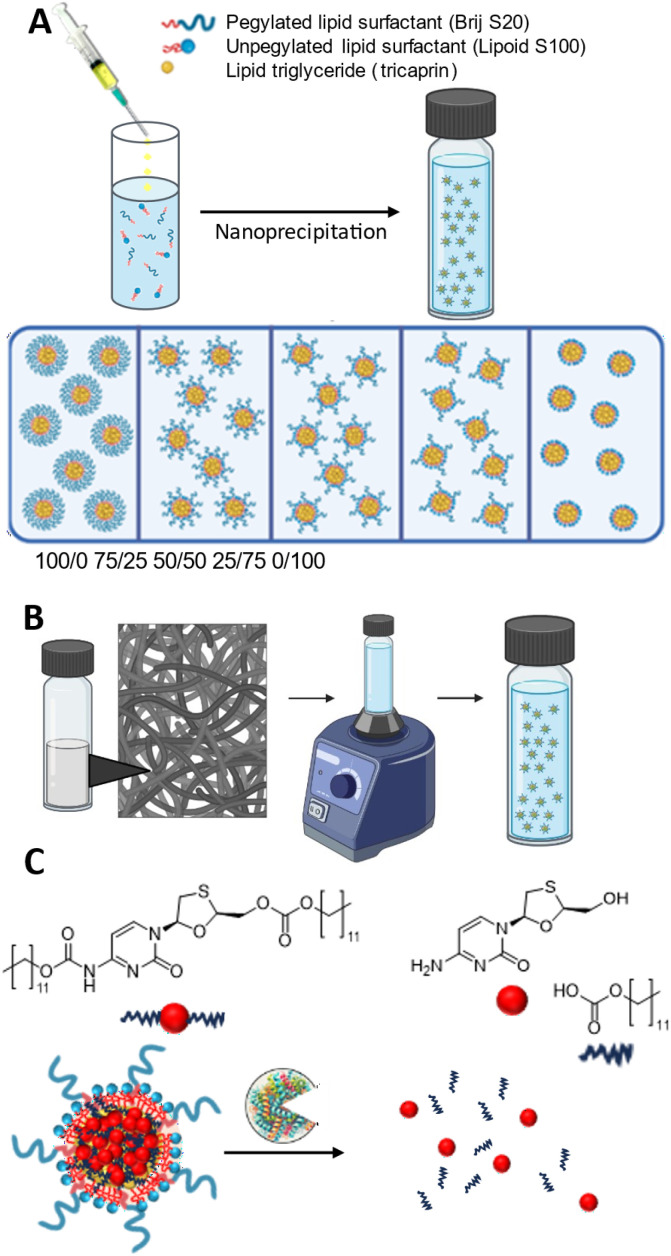
Development of enzymatically triggered drug release from a cryopreserved LNP. This work involved three stages: (A): Investigation of the role of surfactant composition (PEGylated *vs.* unPEGylated) on the properties of the LNPs. (B): Evaluation of the influence of surfactants and cryoprotectant concentrations on the formation of a formulation that can be successfully lyophilised and redispersed. (C): Production of LNPs through the incorporation of a prodrug, demonstrating its activation and release of 3 TC in the presence of an enzyme.

This work develops drug-loading LNP formulations, using a bis-prodrug, that are compatible with cryopreservation and capable of enzymatic drug release. We systematically investigated how formulation properties, such as surfactant composition and core material weight percentage (wt%), influence the ability of LNPs to withstand cryopreservation stresses. Using a combination of pegylated (Brij S20) and unpegylated, zwitterionic (Lipoid S100) lipid surfactants, we optimised their ratios to enhance colloidal stability and redispersibility (Fig. 1A). Formulation stability was assessed across a range of core material loadings (14–40 wt%), identifying the optimal balance between drug loading and cryostability. A hydrophobically modified lamivudine bis-prodrug was incorporated into the most promising formulation to investigate its potential for drug loading and enzymatic activation (Fig. 1B). Finally, *in vitro* drug release studies demonstrated sustained prodrug activation in the presence of porcine liver esterase, confirming release and enzymatically triggered prodrug activation (Fig. 1C). In contrast to existing studies that examine lyophilised LNPs or enzyme-responsive systems individually, our study uniquely integrates surfactant ratio variation, cryopreservation behaviour, and enzymatically activated release of a bis-prodrug for a hydrophilic small molecule. This provides a physicochemical framework for designing stable, redispersible LNPs capable of delivering drugs with prolonged release profiles.

## Results and discussion

### Investigating the role of the surfactant plays in cryo-stability

In order to understand how the surfactant composition influenced the ability to form samples that could be lyophilised and redispersed, freeze–thaw experiments were first carried out. This approach was used to identify how the compositions of the two surfactants-controlled stability during the freezing stage.^11,43^

The lipid used in this work was tricaprin, a triglyceride which has shown to be versatile due to its use in a wide range of LNP formulations.^8,35,44,45^ LNPs were produced by nanoprecipitation of the tricaprin dissolved in the organic solvent tetrahydrofuran (THF) into an aqueous solution (aqueous/organic ratio of 12/1) with different surfactant compositions (PEGylated (Brij S20) *vs.* unPEGylated (Lipoid S100)) at ratios from 100/0, 75/25, 50/50, 25/75 and 0/100. From hereon, the sample formulation will be described in terms of their surfactant composition based on the ratio Brij S20/Lipoid S100. Brij S20 (polyoxyethylene 20 stearyl ether) is a pegylated lipid derived from stearic acid molecules conjugated PEG (1000 g mol^−1^). Lipoid S100 is a zwitterionic molecule derived from soybean and has a phosphatidylcholine content ≥94%. These surfactants were selected to provide different particle stabilisation methods steric (Brij S20) or hydration repulsion which is the repulsive interactions provided by zwitterionic surfactants (Lipoid S100).^46^ After nanoprecipitation the formulations were kept in unsealed containers for two days to allow for the THF to evaporate, we have previously shown that no THF could be detected after this duration.^35^

**Fig. 2:**
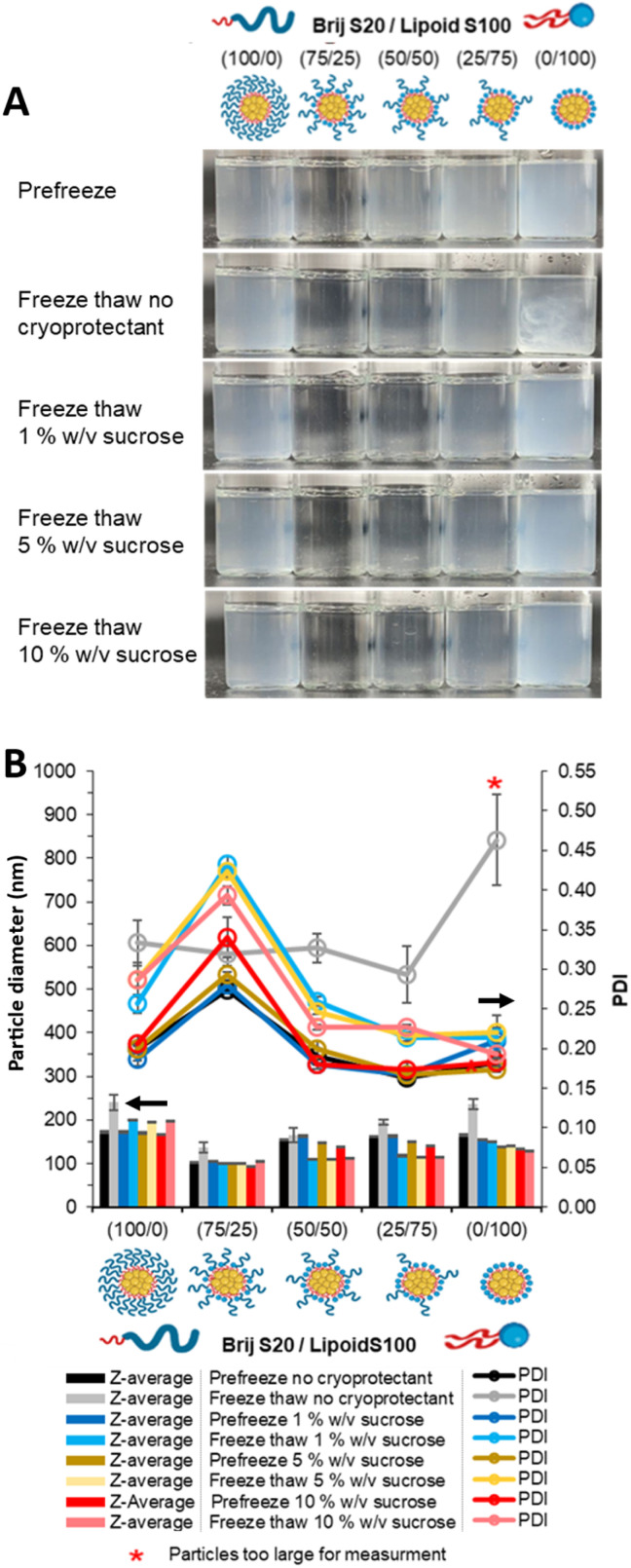
Effect of cryoprotectant concentration on the formulations of tricaprin at 14 wt% with varied in pegylated/unpegylated lipid surfactant blends after freeze–thaw on Day 2 after formulation. (A) Photos of each formulation before freeze thaw and after freeze thaw with different concentrations of cryoprotectant (no cryoprotectant, 1, 5 and 10% w/sucrose). (B) The z-average diameter and PDI of each of the samples as measured by DLS at ∼0.58 mg mL^−1^ excluding sucrose.

Prior to freeze–thaw the most turbid samples were those stabilised by a single surfactant *i.e.* 0/100 followed by 100/0. The least turbid samples had a surfactant composition of 75/25 (Fig. 2A). The different formulations were rapidly cooled with liquid N_2_ to freeze both the water and THF after the addition of aqueous sucrose solutions (previously shown to be an effective cryoprotectant for nanoparticle dispersions^47^) to give cryoprotectant concentrations of 0, 1, 5 and 10% w/v of in the final formulation. Then after thawing, the visual appearance of each formulation was observed (Fig. 2A). This experiment therefore provided an insight into the effect of the two different surfactants and the influence of the cryoprotectant on the formulation stability after freezing and thawing. When no cryoprotectant was used, the formulation solely comprised of 0/100 (100% unpegylated lipid surfactant), possessed a clearly observable pearlescence, indicative of micron-sized anisotropic crystals likely the result of aggregation (confirmed by optical microscopy as shown in Fig. S1). All the other formulations (those with ≥25% pegylated surfactant) did not display any visual changes that would indicate colloidal instability. Formulations containing 1% w/v sucrose as cryoprotectant had a visual appearance similar to the formulation pre-freezing, with the exception that the 0/100 formulation (100% Lipoid S100) was more turbid, potentially indicating larger particles due to an increase in light scattering. At concentrations of sucrose from 2–10% w/v it was not possible to visually observe a difference between the formulations that had undergone freeze–thaw compared to the formulation pre-freezing.

To provide a more detailed understanding of characteristics of the samples, each formulation was characterised, using electrophoretic mobility measurement to determine zeta potential and dynamic light scattering (DLS) to determine the z-average hydrodynamic diameter and polydispersity index (PDI). Prior to freezing all formulations possessed zeta potential values between −4.2 and −6.0 mV (Table S1), as would be expected for the two surfactants neither of which possessed a net charge. All the formulations also had similar mean diameters of 155–170 nm and PDI between 0.17-0.22, except for the 75/25 formulation which was smaller (105 nm) and more polydisperse (0.29) (Fig. 2B). Given that smaller particles have weaker scattering, this explains why this formulation also had the lowest turbidity (Fig. 2A). The z-average diameter range of our LNPs are similar to those previously reported that use tricaprin as the predominate lipid in the core, Din *et al.* showing mean diameters in the range 138–189 nm but produced by high pressure homogenisation.^48^ After freeze–thaw, when no cryoprotectant was used there was a marked increase in mean diameter and PDI for all samples, with formulations containing less pegylated lipid surfactant (Brij S20) tending to be larger. The anisotropic crystals observed for 0/100 formulation produced a DLS correlation coefficient curve of poor quality indicating the DLS data for this formulation was unreliable. We have shown it is possible to prevent particle aggregation using lower concentration of cryoprotectant compared to prior findings on LNPs where concentration of at least 10% or 12% were required.^11,49^ Overall, the visual observation of the formulations proved to be an effective way of identifying formulations which did not tolerate freeze–thaw and showing the need for sucrose as a cryoprotectant to enable all formulations to remain colloidally stable after freeze–thaw.

**Fig. 3:**
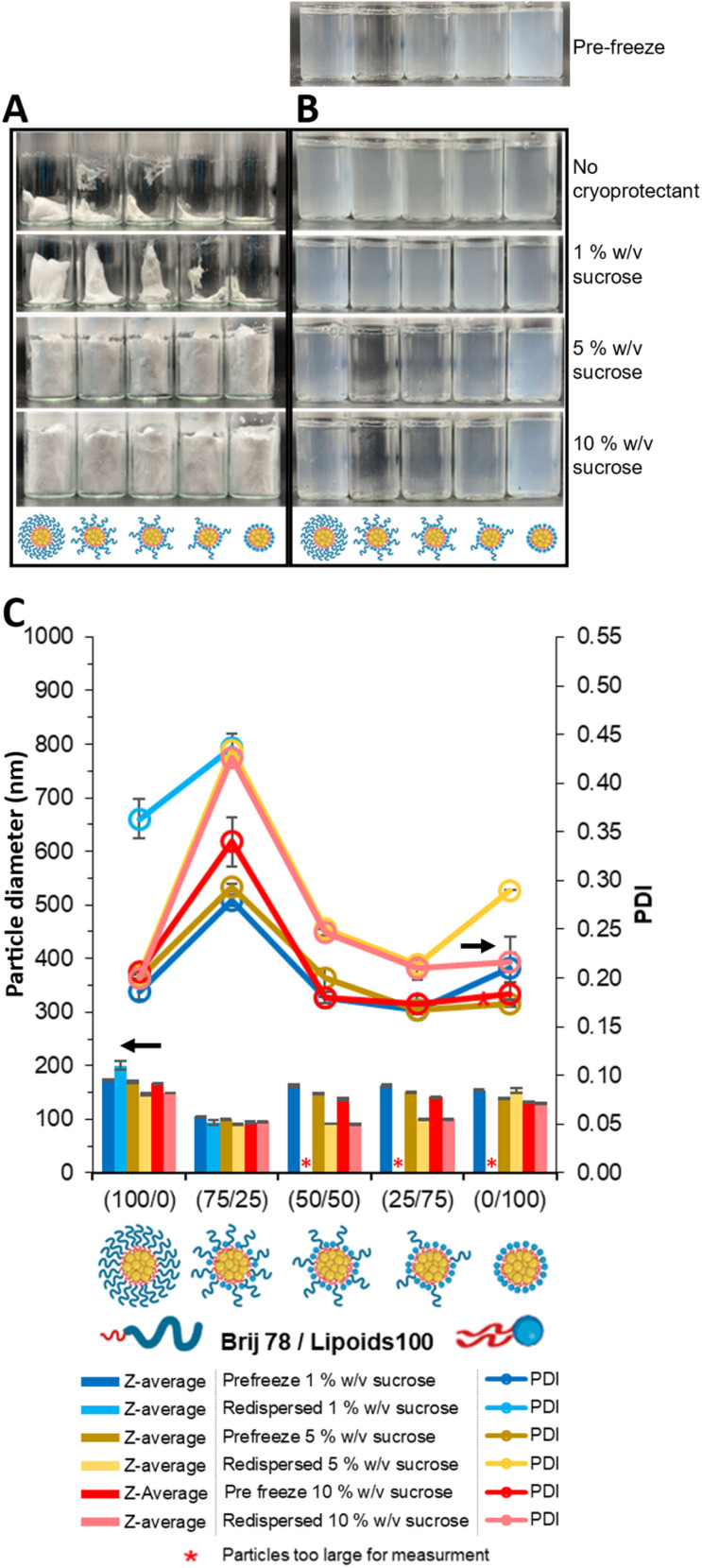
Effect of cryoprotectant concentration and surfactant composition on appearance of the formulations after freeze–drying and after redispersion samples were freeze-dried on Day 2 after formulation. (A) Photos of each formulation's cake once freeze-dried with different concentrations of cryoprotectant (sucrose) as well as surfactant composition. (B) Photos of each of the same formulation before and after lyophilization. (C) Z-average diameter, and PDI obtained by DLS. All formulations contained tricaprin at 14 wt% with varied in pegylated/unpegylated lipid surfactant blends on a mass ratio. Measurements made at a fixed position of 4.65 mm in the DLS at ∼0.58 mg mL^−1^ excluding sucrose.

The same series of samples were subjected to lyophilisation to assess the ability to withstand the stresses of sublimation as well as freezing. As previously, the formulations were characterised by visual observation after lyophilisation (Fig. 3A) then upon dispersion in water (Fig. 3B). The form of each dried formulation can be seen in Fig. 3A. There was a noticeable trend as the ratio of pegylated lipid was reduced the cake size decreased, with the lyophilised material formulation with 0/100 (100% Lipoid S100) presenting as a sticky layer on the bottom of the vial. Furthermore at 1% w/v sucrose there was a noticeable degree of cake collapse, however cakes appeared to maintain integrity at 5 and 10% w/v sucrose. Cake integrity and stability has previously been shown to be essential to ensure efficient redispersion of a formulation.^20,50^ Furthermore, for each formulation there was an increase in the size of the cake produced with increased concentration of sucrose as the percentage of sucrose in the total formulation also increased (the compositions of the formulations can be seen in Table S2). The dried formulations were then redispersed (Fig. 3B). Without any cryoprotectant all the redispersed formulation displayed pearlescence like that shown in Fig. 2A, indicating the presence of anisotropic particles in suspension likely the result of aggregation. At 1% w/v sucrose, formulations stabilised by 50/50, 25/75 and 0/100 also showed signs of aggregation with pearlescence increasing with increasing Lipoid S100 composition. However, formulations stabilised by a high proportion of Brij S20 appeared stable and showed no clear sign of large particles.

Characterisation of the dispersions by DLS (Fig. 3C) showed that the increased turbidity of the samples seen in the visual observation generally corresponded to larger z-average hydrodynamic diameters. When 1% w/v sucrose cryoprotectant was used, only the dispersions with 100% pegylated surfactant (Brij S20) were suitable for DLS analysis, *i.e.* the other formulations generated error reports in the DLS software due to the samples being too large and/or polydisperse for DLS measurement. For the other formulations, the size distributions of the formulations were generally monomodal (Fig. S2–4) meaning that the mean diameters can be used to compare the formulations. When ≥5% wt sucrose was used as the cryoprotectant then all composition of surfactant produced dispersion with mean diameters between 90–170 nm. This suggests the pegylated surfactant Brij S20 provided necessary steric stabilisation to prevent/limit aggregation of nanoparticles. Brij S20 likely aided in cake formation and prevented collapse, enhancing redispersibility. De Chasteigner *et al.* demonstrated that hydrophilic surfactants are essential for nanoparticle stability post-freeze drying and redispersion.^51^ Therefore, Brij S20 significantly influenced LNPs formulation redispersibility. When Brij S20 was used predominantly (100/0 or 75/25), formulations were redispersible with sucrose concentrations of 1% wt, otherwise, adding sucrose at ≥5% w/v as a cryoprotectant was necessary. Additionally, at these higher sucrose concentrations cake collapse did not occur and the combinations of the pegylated surfactant (Brij S20) with the unpegylated, zwitterionic surfactant (Lipoid S100) at all compositions (75/25, 50/50 and 25/75) showed smaller mean diameters likely due to the combination of steric and hydration repulsion provided by the two surfactants respectively.

### Obtaining API loaded formulations

The lipid core of LNPs is the carrier within the particles that encapsulates the API. Therefore, to increase the potential API loading capacity of the formulations, the amount of the lipid, tricaprin was increased from 14% to 33% or 40% tricaprin in the LNP formulation. This increase was achieved by increasing the concentration of tricaprin in the THF phase during nanoprecipitation.

To allow for the production of a sample that could be redispersed after freeze drying a cryoprotectant concentration of 10 wt% was selected as it had produced samples with the lowest PDIs in the prior study. The formulations containing the increased tricaprin with 10 wt% sucrose were then lyophilised, redispersed and analysed by DLS to understand the effect of surfactant composition on particle properties (the compositions of the formulations are shown in Tables S3 and S4). Each formulation with 40% tricaprin produced a pearlescent dispersion and the presence of anisotropic crystals was supported by optical microscopy, Fig. S5. It is hypothesised that increasing the core material may have meant that there was too much nanoparticle surface area to achieve effective surface coverage by surfactant. This reduction in stabilisation could lower the barrier to nanoparticle aggregation preventing the formation of nanoparticles from nanoprecipitation.

**Fig. 4:**
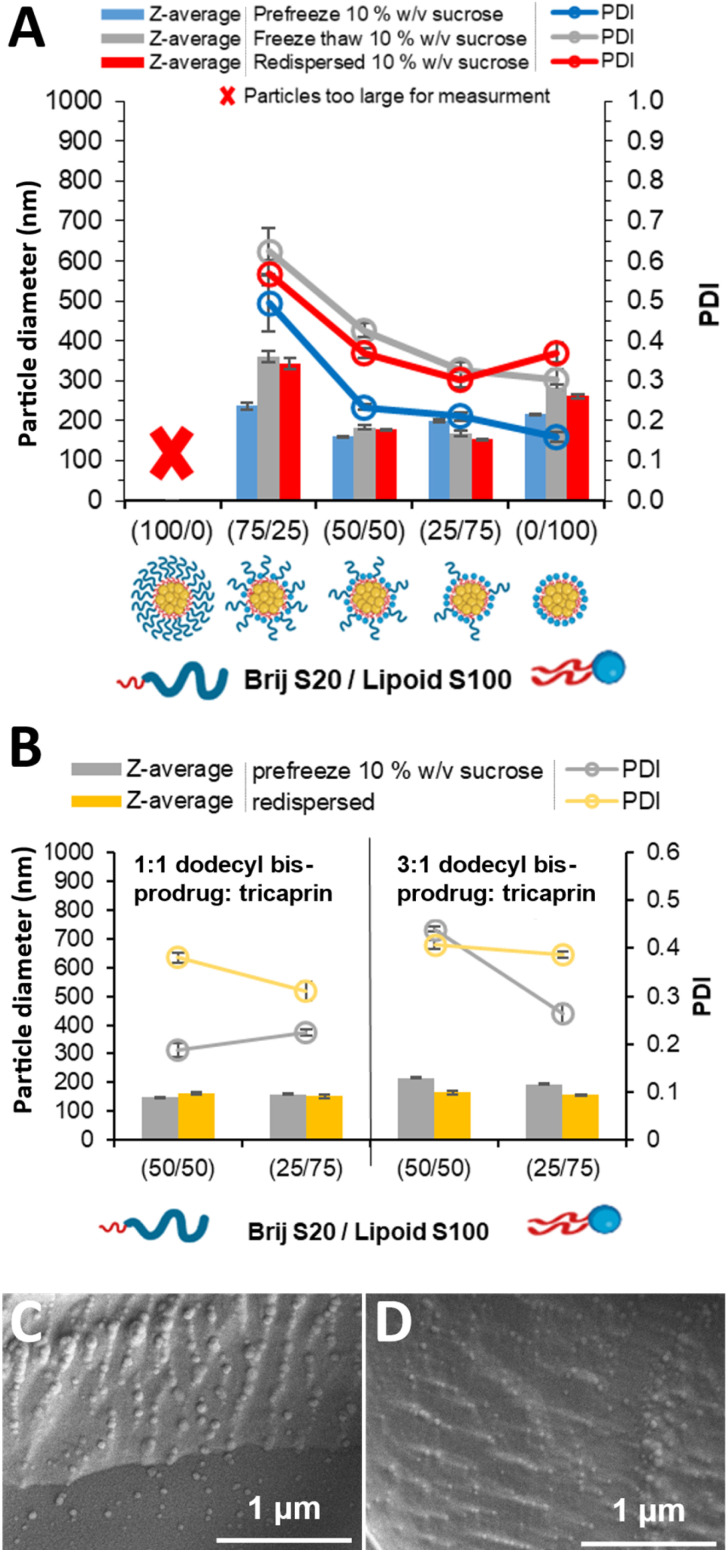
Characterisation of the formulations of tricaprin at 33 wt% varied in pegylated/unpegylated lipid surfactant blends on a mass ratio. (A) Z-average particle diameter and PDI of the different surfactant formulations, prefreezing, after freeze thaw, and after freeze drying and redispersion as measured by DLS with 10% w/v of sucrose cryoprotectant. (B) Z-average particle diameter and PDI as measured by DLS and formulations made of dodecyl prodrug/tricaprin blends at an overall 33 wt% varied in pegylated/unpegylated lipid surfactant blends on a mass ratio. (C and D) CryoSEM images of the formulation at 33 wt% with a core composition of 50/50, 3 : 1, while also in the presence of 10% w/v sucrose. Before freeze drying (C) and redispersed after freeze drying (D).

With the slightly lower tricaprin content of 33% tricaprin, nanoscale dispersions were formed at all surfactant compositions other than 100/0. Fig. 4A displays the particle mean diameter and PDI data for formulations at 33% tricaprin. The dispersion stabilised by 75/25 displayed an average increase in mean diameter by ∼125 nm and increase in PDI upon freeze–thaw and lyophilization, indicated a degree of aggregation induced by the freezing stage. Additionally, the 0/100 formulation also displayed a slight increase in mean diameter and an increase in PDI from ∼0.15 to 0.35 likely also due to aggregation. During the freezing of nanoparticles, the particles are concentrated in the cryoconcentrated phase which may cause their aggregation or fusion.^52^ We have previously shown that Lipoid S100 can likely participate in LNP nanoparticle core formation.^32^ Interestingly, increases in mean diameter and PDI were found for the freeze–thaw stage of the process and no further increase in diameter or PDI after lyophilization and redispersion. This behaviour suggests that issues with colloidal stability for these formulations were occurring during the freezing stage, likely through a cryoconcentration process. Different behaviour was seen for the formulations with a surfactant composition of 50/50 or 25/75 which displayed very little change in mean diameter when comparing the pre-freeze diameter with the values found after freeze–thaw or lyophilization and redispersion. Both formulations displayed a slight increase in PDI although these PDI values were all <0.45. We speculate that the combination of steric and hydration repulsion provided by the two surfactants prevents aggregation. Therefore, our findings show the importance of surfactant combination in terms in providing particle stability during freezing.

We have previously demonstrated that hydrophobic prodrug-type modification of the API lamivudine (used to treat human immunodeficiency virus infection) enabled stable nanoparticle formation. When the API was modified to have a high log *P* value (11) the prodrug formed particles of low polydispersity and enhanced colloidal stability due to rapid nucleation and reduced Ostwald ripening.^33^ Building on this, we aimed to produce formulations of a dodecyl bis-prodrug of lamivudine, structure shown in Fig. 1C, (from here-on named bis-prodrug (BPD)) that could be lyophilised and redispersed. To this aim, BPD was blended at ratios of 1 : 1 and 3 : 1 with tricaprin maintaining a 33 wt% core material in the LNP formulation. We also tested these different BPD loadings at the two surfactant composition (50/50 and 25/75) that had shown the best stability during the freezing, lyophilisation and redispersion tests. The naming of the formulations in this study are based on the surfactant composition and then the ratio of BPD to tricaprin, *i.e.* 50/50, 3 : 1 is a formulation of 50% Brij S20, 50% Lipoid S100 with a core of 3 : 1 mass of BPD : tricaprin. To achieve the LNPs formulations, the BPD, tricaprin and Lipoid S100 were dissolved in THF and which was then nanoprecipitated into an aqueous solution of Brij S20. After the THF had evaporated after two days, the resulting LNP formulations had a theoretical composition of 16.7 or 25.0% BPD by mass, which equated to 5.8 or 8.8% API loading, for the 1 : 1 or 3 : 1 BPD:tricaprin formulations respectively. The full theoretical compositions for our formulations are shown in Table S5. Mass loadings of LNPs are frequently not directly reported prior literature articles. However, for a number of different small molecule API drug loading values of 0.05–11% have been reported in LNPs, with the majority of formulations possessing loadings of <5%.^53^ API loadings for single-site modified prodrugs based LNPs of 4% or 11% have been reported previously (see Table S6).^37,54^ Therefore, our loading values are consistent with those reported in the literature for similar LNP systems, indicating that the observed loading is not an inherent limitation of the bis-prodrug approach.

To obtain formulations with cryoprotectant the LNPs were mixed with the sucrose cryoprotectant solution (10 wt%) before being lyophilized and redispersed. This addition of the cryoprotectant resulted in a considerable reduction in the mass percentage of the API in the formulations to 0.2 or 0.4% (full theoretical compositions of the formulations are shown in Table S7). For the API used in our study, lamivudine, typically to daily dose of 300 mg is given daily with a bioavailability of 82% in adults and 68% children.^55^ For our system, given a loading of 0.4%, a 300 mg dose would require 75 g of the full formulation to be administered. The highest dispersion concentration of the full formulation we tested was at 0.48 mg mL^−1^ lamivudine and 120 mg mL^−1^ in terms of the full formulation, at this concentration the formulation looked visually stable and homogeneous (Fig. S10). At this concentration then 75 g of formulation would be dispersed in 625 mL. Clearly, beyond this proof-of-concept work, the focus for future APIs selection should be high potency molecules that would allow for smaller doses.

The resulting dispersions were analysed visually (Fig. S7), all formulations were turbid with no visual particles. Each formulation was characterised by DLS, showing monomodal populations (Fig. S8) and zeta potential values of −5.5 to −7.2 mV (Fig. S9). A comparison of the mean particle diameter and PDI before and after freeze–drying is shown in Fig. 4B. All four of the formulations displayed similar mean diameters of ∼150 nm for the samples with a 1 : 1 ratio of BPD to tricaprin and slightly larger for the formulations with more BPD (3 : 1) ∼160–180 nm. Upon redispersion, all formulations with the exception of 50/50, 3 : 1, displayed an increase in PDI compared to their pre-freeze values, likely due to limited particle aggregation. The improved cryo-stability observed for the 50/50 and 25/75 surfactant ratios likely arises from synergistic effects between steric stabilisation provided by the PEG-containing surfactant and hydration repulsion imparted by the zwitterionic headgroup of Lipoid S100. At intermediate compositions, the mixed corona may better prevent close approach of LNPs during freezing and rehydration, thereby minimising fusion or aggregation compared to formulations with only one stabiliser. CryoSEM was used to characterise the LNPs due to the ability of this technique to image dispersions while avoiding drying artifacts typical for conventional SEM. Fig. 4C shows the 50/50 3 : 1 dispersion pre-freeze displaying the presence of nanoparticles. After dispersion, Fig. 4D spherical particles were also observed. Indicative measurement from the particles observed by cryo-SEM image revealed average diameters of 94 and 81 nm (standard deviations of 28 and 22) for pre-freeze dry and redispersed retrospectively. The particle size distributions can be seen in Fig. S10. However, it is important to note that these images represent fracture planes through a vitrified matrix, and the observed features often include partial voids or impressions left by particles dislodged or bisected during fracturing. This fractures plane can pass through any part of the particle, not necessarily the equator, and therefore these features do not reliably represent the full particle diameter. Consequently, the apparent diameter of these impressions will underestimate the true particle diameter unless an entire particle remains embedded and visible on the fracture surface, but confirms nanoscale features consistent with nanoparticle morphology were observed. This explains why the mean diameters measured by CryoSEM were smaller than those found by DLS (216 and 166 nm before and after freeze drying).

**Fig. 5:**
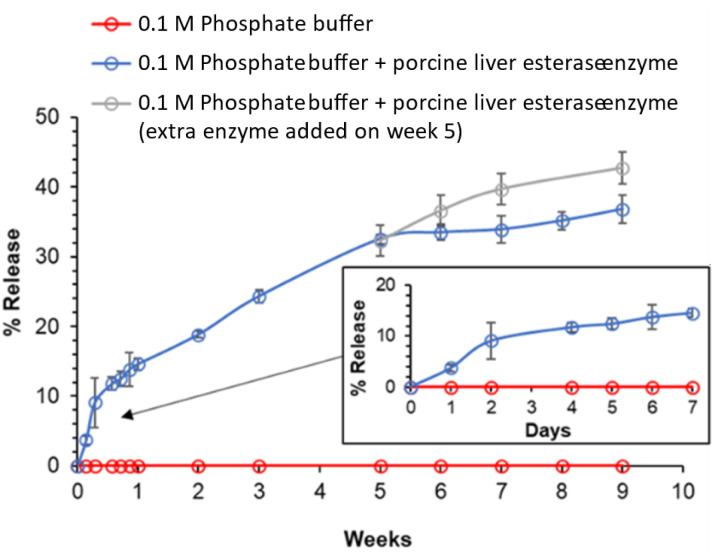
Cumulative release of lamivudine from LNP formulation 50/50, 3 : 1 as determined by HPLC analysis. Drug release was monitored over 9 weeks in the presence or absence of porcine liver esterase enzyme.

The ability for drug release to be triggered by enzymes may provide benefits in terms of controlling the release kinetics. The activation of the prodrug from the LNPs was investigated with formulation 50/50, 3 : 1 (and 10% wt sucrose). This formulation was selected due to its ability to redisperse with ease as well as containing a higher loading of drug compared to the formulations with a 1 : 1 ratio of BPD to tricaprin. The BPD formulation was prepared, freeze dried and concentrated by redispersing into a smaller volume. This increase in concentration of lamivudine was used to ensure that we were able to quantify the presence of the API at percentages of activation ≥0.2% (see Fig. S11 for the HPLC calibration data). These concentrated dispersions then treated with porcine liver esterase enzyme and compared to a control sample where no enzyme was present. Porcine liver esterase was selected as a broadly active *in vitro* esterase that has been widely used in prodrug release studies. Its diverse isoenzyme profile provides a more representative enzymatic environment than individual recombinant carboxylesterases, and its activity has been shown to represent human tissue esterases in prior studies.^54,56–58^ DLS was not applicable to monitor size reduction throughout the release experiment due to the high sucrose concentration, visual observation of the samples over the duration of the experiment indicated that no aggregation of the LNPs had occurred. At frequent timepoints aliquots of the sample were removed, quenched with methanol to prevent further enzyme activity and the soluble components separated from the particles using a 10 kDa membrane. The resulting filtrate was analysed by HPLC against a calibration curve for lamivudine. Thus, this approach allowed the quantification of the activation of the BPD to release the API (lamivudine). The formation of lamivudine over time is shown in Fig. 5. When no enzyme was used (PBS alone) then lamivudine no detected in the release media. In the presence of enzyme, ∼4% of total initial theoretical loaded API was detected after 24 hours, ∼15% was measured after one week and ∼37% cumulative release after 9 weeks, Fig. 5. To further confirm that the compound being detected was indeed, the activated API, a sample from week 9 was also analysed by HPLC-MS which found 230.05 [M + H]^+^ for the peak at ∼0.85 minutes, thus indicating the presence of lamivudine. Between weeks 1–5 the difference in drug release was ∼18% over four weeks translating to ∼5% per week. There was a linear relationship of drug release over weeks 1–5. After week 5, the rate of release began to reduce, with an average release of 4% released between weeks 5 and 9 (∼1% per week). The reduction of drug release rate may be explained by irreversible enzyme inhibition such as suicide inhibition of the enzymes, whereby a degradation product irreversibly binds to the enzyme by forming a covalent bond.^59^ Indeed, Yan *et al.* have highlighted potential suicide inhibition occurrence in esterases in the presence of sugars such as sucrose.^60^

After week 5 sampling, formulations with enzyme were split: one set kept the original enzyme concentration, and the other tested the effect of fresh enzyme, as shown in Fig. 5. The addition of fresh enzyme resulted in greater drug release than the samples that only contained the original enzymes, with ∼43% drug release measured by week 9 when the experiment was stopped. This data further suggests that the enzyme than had been added at the start of the experiment had begun to lose activity. It is plausible to assume ∼100% drug release could hypothetically be achieved upon further additions of fresh enzyme. We believe that the mechanism for the activation of the BPD is likely a combination of two behaviours: 1. Despite the BPD having a high Log *P*, some BPD will dissolve into the aqueous phase and form a saturated solution. This soluble BPD will then be accessible to be enzymes and be activated to form the lamivudine API. The dramatically increased solubility of the API (>270 mg mL^−1^)^61^ provides a driving force for further dissolution of BPD. At the same time, the hydrolysis of the carbamate and carbonate linkages will yield dodecanonic acid 2. The enzyme is able to access the BPD molecules on the surface on the LNPs and hydrolyse the bonds to release the API, the diffusion of BPD molecules from the inside of the particles to the surface then enables the continued activation of the BPD to the API. Based on our proof of concept experiments we are not able to differentiate between these two behaviours. While the sustained release profile suggests prolonged retention of the bis-prodrug, we acknowledge that the absence of DLS characterisation of the LNPs over the 9-week period means that the contribution of nanoparticle stability *versus* prodrug hydrolysis kinetics cannot be fully resolved.

As no API was detected in the absence of the enzyme this showed that the BPD was stable at physiological conditions when encapsulated within this formulation and activation was only seen when enzymes are present. When comparing our findings with those in the literature to the best of our knowledge there are no other studies on bis-prodrugs of lamivudine in nanoparticles. We have previously studied the activation of a bis-prodrug of a nanosuspension of emtricitabine, a HIV drug with a similar chemical structure to lamivudine. However, in this work we did not directly investigate the activation of the API from the bis-prodrug in the form of a nanosuspension but instead monitored the activation of the solubilised bis-prodrug with mixed with liver s9 fraction (containing enzymes) to determine a predicted half-life in plasma. This analysis showed that the bis-prodrug with the most hydrophobic character (octyl modification compared to the dodecyl modification used in this work) had a half-life of ∼10 hours.^36^ Other studies for different APIs with single-site modified prodrugs loaded within nanoparticles show dramatically faster release rates. For example, Shi *et al.* incorporated their prodrug (chloesterol-SN38 prodrug with ester linker) within the membrane of liposomes with a nanoparticle size of 125 nm and showed ∼50% drug release after 12 hours.^54^ Zhang *et al.* have demonstrated a single site heptadecane alkyl modification of camptothecin with a glutathione cleavable linker in LNPs and showed release reaching a plateau at ∼60% after 24 hours.^37^ Additionally, van der Meel showed that an esterase enzyme triggered a 75% release of docetaxel from a heptadecane alkyl-modified ester linked prodrug from LNPs within 24 hours.^38^ Clearly direct comparison with literature data is not appropriate given the numerous differences between the formulations and prodrugs. However, this comparison does illustrate that our formulation provides much slower activation than previously reported behaviour and therefore has potential application as a long-acting formulations. We hypothesise that the dual-site modification of the lamivudine along with its encapsulation within a LNP appears to considerably decrease the rate of API release when compared to other examples in the literature.

## Conclusions

This study demonstrates the successful development of cryopreserved LNP formulations with enzymatically triggered release, addressing key challenges in the field. By systematically optimising the surfactant composition and cryoprotectant concentration, we identified formulations that retained colloidal stability during freeze–thaw and lyophilisation processes, with sucrose concentrations ≥5 wt% proving critical for preventing aggregation and ensuring redispersibility. The incorporation of a hydrophobically modified lamivudine BPD at 25 wt% of the solids in the formulation enabled API loadings of up 8.8% API loading which is comparable with traditional small molecule formulations. The use of cryoprotectant dramatically reduced the mass API percentage within to 0.4%, a penalty associated with cryoprotectant use. Obtaining mass efficient API loadings in lyophilised formulations remains a challenge in the field, this work demonstrates the importance of surfactant selection revealing the influence that combinations of surfactants can assist the formation redispersible LNP systems. Enzymatic activation studies revealed sustained lamivudine release over nine weeks, with activation kinetics modulated by enzyme activity. Clearly, prodrug strategies and particularly bis-prodrug involve a mass penalty in terms of API content within the formulation. However, our findings show that the bis-prodrug approach enables the incorporation of traditionally water-soluble APIs into LNPs formulations, as well as slow activation to form the API that might be of value in long-acting drug formulations.

Additionally, this work provides a versatile platform for addressing the stability and controlled release challenges of LNPs, offering opportunities for their broader application in water-soluble small molecule therapeutics, particularly for diseases requiring slow release of the API. Future studies will focus on expanding this approach to other prodrugs and therapeutic agents, as well as evaluating *in vivo* performance to translate these findings into clinical settings.

## Methods

### Materials

Dodecyl bis-prodrug (dodecyl (1-((2R,5S)-2-((((dodecyloxy)carbonyl)oxy)methyl)-1,3-oxathiolan-5-yl)-2-oxo-1,2-dihydropyrimidin-4-yl) carbamate) was used as synthesised as described in our previous publication.^35^ Phosphate buffered saline, Brij S20 and tetrahydrofuran, and lyophilised porcine liver esterase enzyme, ammonium formate salt, methanol and HPLC grade acetonitrile were all purchased from Sigma-Aldrich and used as received. Lipoid S100 was purchased from Lipoid and used as received. Tricaprin was purchased from Tokyo chemical industry and used as received.

### Methods

#### General nanoparticle preparation

Method adopted for nanoparticle formulation was nanoprecipitation and was derived from a literature approach.^28^ Briefly, the method was as follows, the aqueous phase, a stock solution of Brij S20 was prepared at 1 mg mL^−1^. Portions of the stock solution were taken and potentially diluted further with distilled water to obtain different concentrations of Brij S20; composition shown by Table 1. For the organic phase stock solutions of tricaprin (4 mg mL^−1^) and Lipoid S100 (24 mg mL^−1^) were prepared in tetrahydrofuran, compositions are shown by Table 1. The organic phase was charged dropwise into the vortex of the aqueous phase contained in a 40 mL vial while mechanically stirring (800 rpm). To ensure consistency in time of injection the shot was charged by removing the plunger of a clamped syringe resulting in a steady flow through the hypodermic needle. The combined mixture was left stirring to allow evaporation of tetrahydrofuran over 2 days at a room temperature (∼22 °C) in a fume cupboard with an average air velocity of 0.35 m s^−1^. Samples were then stored at 22 °C.

**Table 1:** Aqueous and organic phase composition depending on surfactant blend. For each sample 24 mL of aqueous phase was used and 2 mL of organic phase

Surfactant composition-ratio of Brij S20/Lipoid S100	Volume Brij S20 stock solution (mL)	Volume distilled water (mL)	Volume tricaprin stock solution (mL)	Volume Lipoid S100 stock solution (mL)	Volume pure THF (mL)
100/0	24	0	1	0	1
75/25	18	6	1	0.25	0.75
50/50	12	12	1	0.5	0.5
25/75	6	18	1	0.75	0.25
0/100	0	24	1	1	0

For the preparation of lipid nanoparticle formulations varying in pegylated lipid Brij S20 and unpegylated lipid Lipoid S100 at elevated wt%, the formulations were prepared in the same way as at 14 wt% although the concentration of the tricaprin stock solution was increased *i.e.* (33 wt%, 12 mg mL; 40 wt%, 16 mg mL^−1^).

For the preparation of lipid nanoparticle formulations varying in pegylated lipid Brij S20 and unpegylated lipid Lipoid S100 at elevated wt%, the general preparation method was the same although composition of organic phase adjusted; Table 2 displays an example of blends of tricaprin and dodecyl drug analogue/prodrug at 40 wt%. Stock solution concentrations; tricaprin (12 mg mL^−1^) and dodecyl drug analogue (12 mg mL^−1^). As the formulation process involves direct solvent evaporation without any filtration or separation steps, all of the bis-prodrug present during formulation is retained in the final dispersion. Therefore, drug loading was calculated theoretically based on input concentrations.

**Table 2:** Organic phase composition depending on blends of tricaprin, dodecyl drug analogue/prodrug as well as surfactant blends

Core composition ratio tricaprin/dodecyl prodrug	Surfactant composition ratio Brij S20/Lipoid S100	Volume tricaprin stock solution (mL)	Volume dodecyl drug analogue stock solution (mL)	Volume Lipoid S100 stock solution (mL)	Volume neat THF (mL)
50/50	50/50	0.5	0.5	0.5	0.5
50/50	25/75	0.5	0.5	0.75	0.25
25/75	50/50	0.25	0.75	0.5	0.5
25/75	25/75	0.25	0.75	0.75	0.25

### Cryopreservation methods

Once THF had evaporated off after 2 days, 2 mL of each formulation was mixed 1 : 1 with sucrose stock solution to achieve a final 1, 5 or 10% w/v sucrose; 2 mL of formulation with mixed with 2 mL of 2, 10 or 20% w/v sucrose stock solution in a 12 mL vial. Vials were then placed into liquid nitrogen for ∼5 minutes until completely frozen. For freeze thaw samples were allowed to thaw out at ∼21 °C. For freeze drying, samples were freeze dried in a VirTis Bench Top K freeze dryer (SP Scientific, Ipswich UK). Condenser temperature was set to −100 °C and vacuum of <40 µbar. All samples remained in the freeze dryer for 72 hours. The composition of the samples is given in Table S2.

### Redispersion method

Samples from Fig. 3 and 4 were hydrated with distilled water using the same volume which entered the freeze dryer to restore concentration of API and surfactant to prefreeze (∼0.58 mg mL^−1^, ∼0.75 mg mL^−1^ respectively). Samples were then redispersed using a Vortex-Genie 2 on setting 5 for 2 minutes.

### Nanoparticle characterisation methods

#### Dynamic light scattering (DLS) and zeta potential measurements

Samples were analysed by DLS using The Malvern ZetaSizer Nano S DLS obtain a Z-average and size distribution (PDI) of nanoparticle dispersion. 2 mL of each sample (1.17–1.7 mg mL^−1^) was measured in standard 3 mL fluorimeter cuvettes with a pathlength of 10 mm. All measurements were carried out at 25 °C with a fixed backscattering angle of 173° using automated setting. Each sample was measured once although formulations were done in triplicate.

Zeta potential was also measured using Malvern ZetaSizer Nano S at 1.17–1.7 mg mL^−1^. Samples were measured using automated settings and samples were measured in triplicate in a Malvern Zetasizer nano series disposable folded capillary cell in 0.1 mM NaCl. All measurements were carried out at 25 °C.

### Scanning electron microscopy (SEM)

Freeze dried materials were loaded onto an aluminium SEM specimen stub (12.5 mm diameter) using carbon adhesive tab before using aluminium solution to coat the rim of the carbon tab. Samples were then left overnight for the aluminium coating to dry. This was followed by coating with gold (EMITECH K550X) with a deposition current of 25 mA for 100 s before imaging. The morphology of the freeze-dried materials was then investigated using a Hitachi S-4800 FE-SEM at 2 and 5 kV.

### Cryogenic scanning electron microscopy (cryo-SEM)

Specimens were prepared by freezing a small volume of sample between two brass rivets, which are plunged into slushed liquid nitrogen. Rivets were transferred to a brass loading shuttle under liquid nitrogen and transferred under a nitrogen atmosphere to a preparation stage cooled to −120 °C. Anti-contaminator in the preparation stage was run at −190 °C. Fracture surfaces were created in the frozen specimen by pushing-off the upper rivet from the one held in the shuttle (using a liquid nitrogen cooled knife). The specimens were sublimed for 3 minutes oat −90 °C, to create a contrast mechanism. Fracture surfaces were coated with Pt in the preparation chamber, to make them conductive and the specimens transferred to a cooled stage in the SEM (at −160 °C, with an anti-contaminator held at −190 °C). The specimens were photographed using an in-chamber secondary electron detector using either 1.5 keV or 10 keV and a beam current of 15 pA. Particles diameters were determined by measuring at least 150 particles using ImageJ.

### HPLC

HPLC analysis was performed on a Hypersil gold C_18_ column (50 × 4.6 mm 3 µm) using the following method: 95% solvent A 5% solvent B for 30 seconds before switching to 5% solvent A 95% solvent B over 1 minute before holding at 5% solvent A 95% solvent B for a further 6 minutes at a flow rate of 2 mL min^−1^ (solvent A: 20 mM ammonium formate aqueous solution prepared in distilled water, solvent B: acetonitrile). For calibration plot various concentrations of drug were dissolved in 10 : 1 methanol: 20 mM ammonium formate solution and run using same method as above. Samples were monitored on HPLC at a UV detection of 270 nm.

### HPLC-MS

Samples were analysed using the same HPLC method as above. HPLC-MS was performed on an Agilent 1290 Infinity II UHPLC coupled to Agilent 6540 UHD Accurate-Mass Q-TOF LC/MS with electrospray ionisation source.

### *In vitro* drug release

The formulation was prepared multiple times for a total volume of 288 mL which was then diluted 1 : 1 with 20% w/v sucrose and freeze dried in volumes of 8 mL in 12 mL vials over 4 days using a VirTis Bench Top K freeze dryer (SP Scientific, Ipswich UK). Condenser temperature was set to −100 °C and vacuum of <40 µbar. All samples remained in the freeze dryer for 96 hours. The contents of each vial were reconstituted immediately using 1 mL 0.1 M phosphate buffered saline (PBS) solution per vial. Redispersion was achieved using a Vortex-Genie 2 on setting 5 for 10 minutes. 12 vials each containing 1 mL (1.5 mg mL^−1^ BPD translating to 0.48 mg mL^−1^ lamivudine) redispersed formulations were then combined. From which 12 mL was charged to a 14 mL vial along with porcine liver esterase enzyme. Sample preparation was repeated in triplicate with and without enzyme. This 12 mL volume of the release reservoir was selected to ensure that sink conditions would be maintained throughout the release experiment for lamivudine. For the samples treated with of enzyme, 130 mg (2465 U) of enzyme was used. Vials were continuously and vigorously shaken at 500 rpm in an incubator shaker with a fixed incubation temperature of 37 ± 0.5 °C. 0.2 mL aliquots were collected and quenched with 0.4 mL ice cold methanol then centrifuged at 150 000×*g* for 1 hour using 0.5 mL regenerated cellulose membrane spin filters with a molecular weight cut off of 10 kDa. The filtrate was then analysed by HPLC analysis as described. *In vitro* release experiments were performed in triplicate. Percentage release was determined based on initial amount added to the formulation.

## Conflicts of interest

There are no conflicts to declare.

## Supplementary Material

NA-008-D5NA00675A-s001

## Data Availability

Supplementary information: detailed experimental formulations used across all freeze–thaw and cryopreservation studies, including full compositional tables for each lipid and surfactant ratio, zeta potential measurements, and particle size distributions before and after cryopreservation. It also includes additional microscopy, photographic, and HPLC data to support interpretation of formulation stability, aggregate formation, and drug release. See DOI: https://doi.org/10.1039/d5na00675a.
