# Supplementary material for: Bis-prodrug cryopreserved lipid nanoparticles with enzymatically triggered release
Source: Nanoscale Adv. 2026 Jan 8;8(3):1042–53. doi: 10.1039/d5na00675a (PMC12781925; doi:10.1039/d5na00675a)
Supplement: NA-008-D5NA00675A-s001 [file NA-008-D5NA00675A-s001.pdf]

Supplementary Information (SI) for Nanoscale Advances.
This journal is © The Royal Society of Chemistry 2025

Supporting Information

Bis-prodrug cryopreserved lipid nanoparticles with enzymatically triggered release

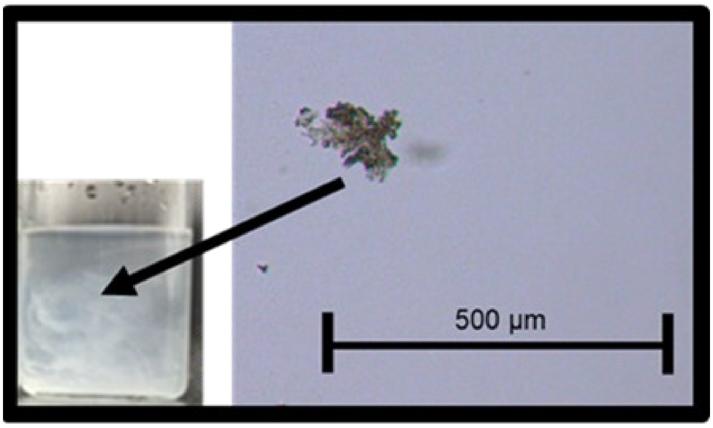

Figure S1. Enlarged image of formulation displaying a slight glittery effect of formulation composed of a surfactant composition of 0/100 (100 % unpegylated lipid) without any cryoprotectant present after freeze thaw including an optical microscope image confirming the presence of crystals because of aggregation due to stress of freezing.

Table S1. The zeta potential measurements on day 2 of formulation (before freezing) of tricaprin formulations measured at 14 wt% for each surfactant composition of Brij S20 and Lipoid S100. Measured in 1 mM NaCl.

**Surfactant Ratio (Brij S20 / Lipoid S100)**	**Zeta Potential) [mV]**	**Standard deviation [mV]**
**100/0**	-6.0	1.0
**75/25**	-4.5	1.3
**50/50**	-5.5	1.1
**25/75**	-5.5	1.4
**0/100**	-4.2	1.2

Table S2. The composition of the samples within the freeze-thaw and cryopreservation experiments. The total 4 ml of sample was prepared using 2 ml of the LNP formulation and 2 ml of sucrose stock solution.

**Formulation**	**Sucrose concentration (% w/v)**	**total mass (mg)**	**Brij s20 (%)**	**Tricaparin (%)**	**Lipoid S100 (%)**	**Sucrose (%)**
100/0	0	2	85.7	14.3	0.0	0.0
100/0	2	42	4.7	0.8	0.0	94.5
100/0	10	202	0.7	0.2	0.2	98.8
100/0	20	402	0.2	0.1	0.2	99.4
75/25	0	2	64.3	14.3	21.4	0.0
75/25	2	42	3.5	0.8	1.2	94.5
75/25	10	202	0.7	0.2	0.2	98.8
75/25	20	402	0.4	0.1	0.1	99.4
50/50	0	2	42.9	14.3	42.9	0.0
50/50	2	42	3.5	0.8	1.2	94.5
50/50	10	202	0.5	0.2	0.5	98.8
50/50	20	402	0.2	0.1	0.2	99.4
25/75	0	2	21.4	14.3	64.3	0.0
25/75	2	42	1.2	0.8	3.5	94.5
25/75	10	202	0.2	0.2	0.7	98.8
25/75	20	402	0.1	0.1	0.4	99.4
0/100	0	2	0.0	14.3	85.7	0.0
0/100	2	42	0.0	0.8	4.7	94.5
0/100	10	202	0.0	0.2	1.0	98.8
0/100	20	402	0.0	0.1	0.5	99.4

Table S3. Formulations with an increased lipid content (40% Tricaprin) in the LNP formulation. The total 4 ml of sample was prepared using 2 ml of the LNP formulation and 2 ml of sucrose stock solution.

**Formulation**	**Sucrose stock solution concentration (% w/v)**	**total mass (mg)**	**Brij s20 (%)**	**Tricaparin (%)**	**Lipoid S100 (%)**	**Sucrose (%)**
100/0	0	3	60.0	40.0	0.0	0.0
100/0	2	43	4.6	3.1	0.0	92.3
100/0	10	203	0.7	0.7	0.2	98.4
100/0	20	403	0.2	0.3	0.2	99.2
75/25	0	3	45.0	40.0	15.0	0.0
75/25	2	43	3.5	3.1	1.2	92.3
75/25	10	203	0.7	0.7	0.2	98.4
75/25	20	403	0.4	0.3	0.1	99.2
50/50	0	3	30.0	40.0	30.0	0.0
50/50	2	43	3.5	3.1	1.2	92.3
50/50	10	203	0.5	0.7	0.5	98.4
50/50	20	403	0.2	0.3	0.2	99.2
25/75	0	3	15.0	40.0	45.0	0.0
25/75	2	43	1.2	3.1	3.5	92.3
25/75	10	203	0.2	0.7	0.7	98.4
25/75	20	403	0.1	0.3	0.4	99.2
0/100	0	3	0.0	40.0	60.0	0.0
0/100	2	43	0.0	3.1	4.6	92.3
0/100	10	203	0.0	0.7	1.0	98.4
0/100	20	403	0.0	0.3	0.5	99.2

Table S4. Formulations with an increased lipid content (33% Tricaprin) in the LNP formulation. The total 4 ml of sample was prepared using 2 ml of the LNP formulation and 2 ml of sucrose stock solution.

Formulation	Sucrose stock solution concentration (% w/v)	total mass (mg)	Brij s20 (%)	Tricaparin (%)	Lipoid S100 (%)	Sucrose (%)
100/0	0	3	66.7	33.3	0.0	0.0
100/0	2	43	4.7	2.3	0.0	93.0
100/0	10	203	0.7	0.5	0.2	98.5
100/0	20	403	0.2	0.2	0.2	99.3
75/25	0	3	50.0	33.3	16.7	0.0
75/25	2	43	3.5	2.3	1.2	93.0
75/25	10	203	0.7	0.5	0.2	98.5
75/25	20	403	0.4	0.2	0.1	99.3
50/50	0	3	33.3	33.3	33.3	0.0
50/50	2	43	3.5	2.3	1.2	93.0
50/50	10	203	0.5	0.5	0.5	98.5
50/50	20	403	0.2	0.2	0.2	99.3
25/75	0	3	16.7	33.3	50.0	0.0
25/75	2	43	1.2	2.3	3.5	93.0
25/75	10	203	0.2	0.5	0.7	98.5
25/75	20	403	0.1	0.2	0.4	99.3
0/100	0	3	0.0	33.3	66.7	0.0
0/100	2	43	0.0	2.3	4.7	93.0
0/100	10	203	0.0	0.5	1.0	98.5
0/100	20	403	0.0	0.2	0.5	99.3

Table S5. Percentage composition of the formulations containing dodecyl bis-prodrug (BPD) and tricaprin targeting at 33% composition in the LNP.

formulation	Brij s20 (%)	Tricaparin (%)	BPD (%)	Lipoid (%)	% of formulation that is API
50/50, 1:1	33.3	16.7	16.7	33.3	5.8
25/75, 1:1	16.7	16.7	16.7	50.0	5.8
50/50, 1:3	33.3	8.3	25.0	33.3	8.8
25/75, 1:3	16.7	8.3	25.0	50.0	8.8

Table S6. Calculation of API loadings for prodrug containing LNP formulations in the literature.

**Publication title**	**Components**	**Mass of lipid (mg)**	**Mass of stabiliser (mg)**	**Mass of prodrug (mg)**	**Mass % of API in the prodrug**	**% of the formulation that is API**
An esterase-activatable prodrug formulated liposome strategy: potentiating the anticancer therapeutic efficacy and drug safety.[1]	**Stabiliser-** 1,2-distaroyl-sn-glycero-3-phosphoethanolamine-N-[methoxy(polyethylene glycol)2000,<br><br>**Lipid-**<br>egg phosphatidylcholine,<br><br>**Prodrug-**<br>SN38-cholesterol with ester linker.	11.61	2	4.38	45.6	11.1
Glutathione-Responsive Nanoparticles of Camptothecin Prodrug for Cancer Therapy.[2]	**Stabiliser-**<br>1,2-distaroyl-sn-glycero-3-phosphoethanolamine-N-[methoxy(polyethylene glycol)2000,<br><br>**Prodrug-**<br>Camptothecin-stearate with disulfide linker	-	10	1	43.8	4.0

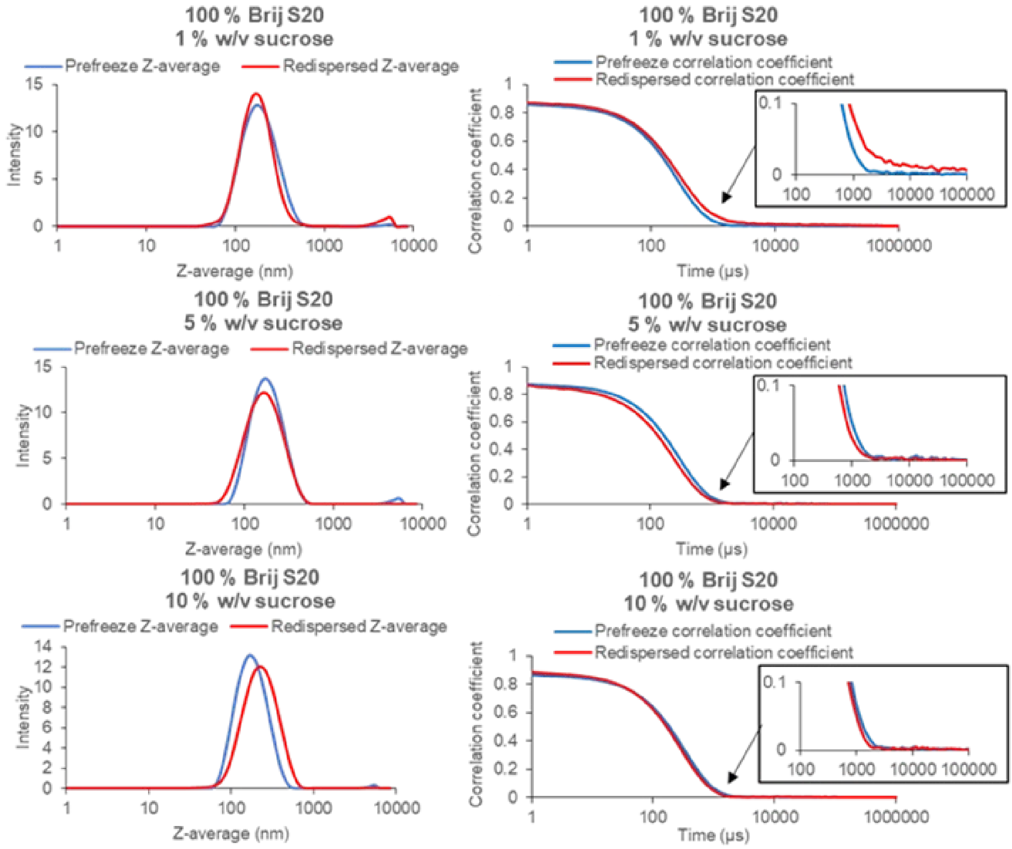

Fig. S2. Particle size distribution graphs and correlation coefficient graphs before and after freeze drying and redispersion for 14 wt % tricaprin formulations stabilised by both 100/0 at 1, 5 and 10 % w/v sucrose. Overall displays how the stability of formulations increases with increasing sucrose concentration.

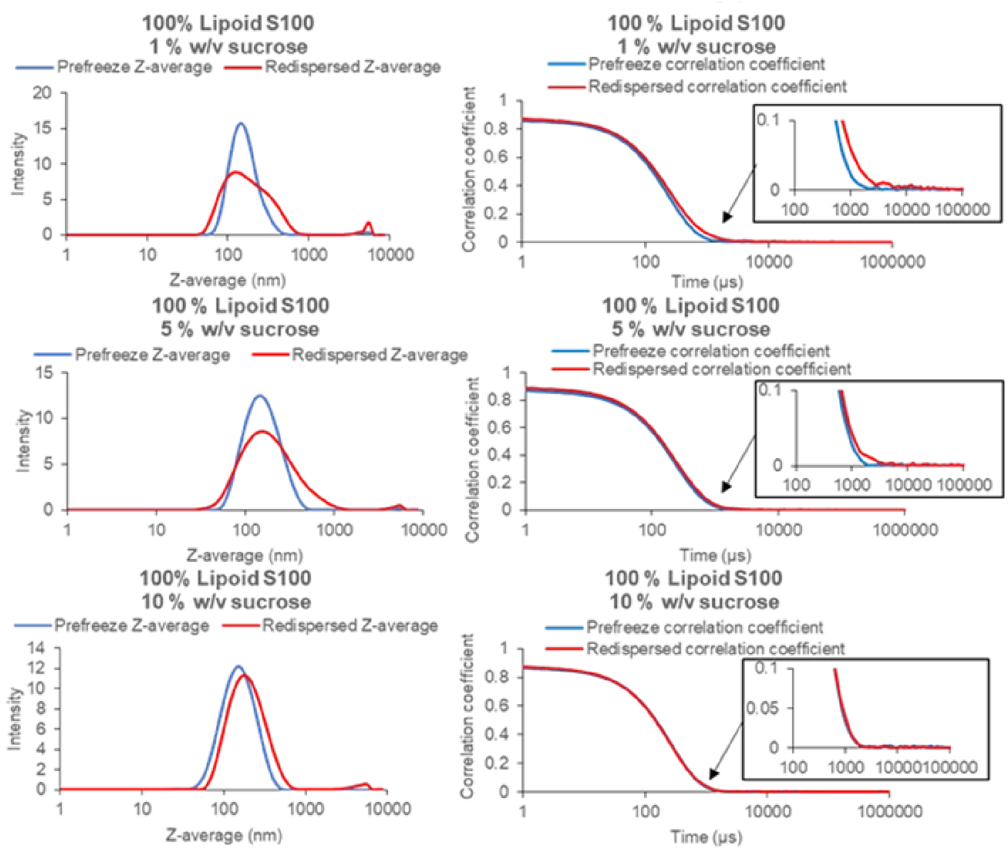

Fig. S3. Particle size distribution graphs and correlation coefficient graphs before and after freeze drying and redispersion for 14 wt % tricaprin formulations with surfactant compositions of 0/100 at 1, 5 and 10 % w/v sucrose. Overall displays how the stability of formulations increases with increasing sucrose concentration.

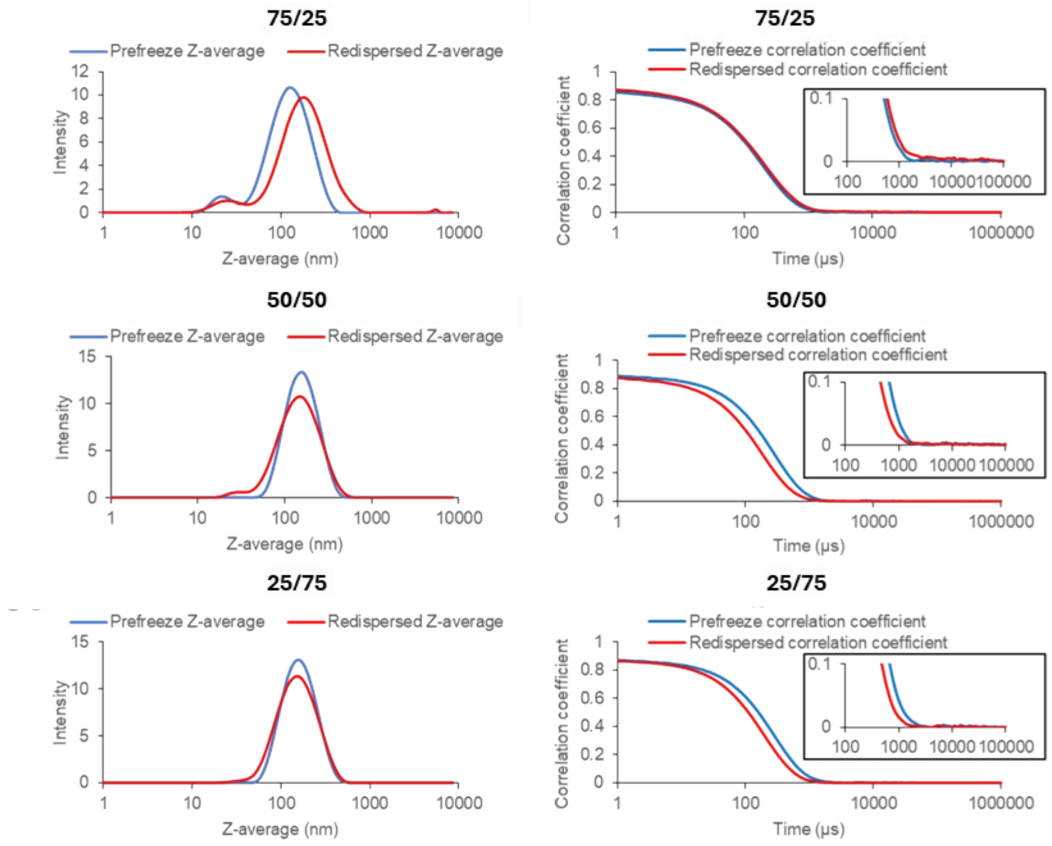

Fig. S4. Particle size distribution graphs and correlation coefficient graphs before and after freeze drying and redispersion for 14 wt % tricaprin formulations with surfactant compositions of 75/25, 50/50 or 25/75 at 10 % w/v sucrose.

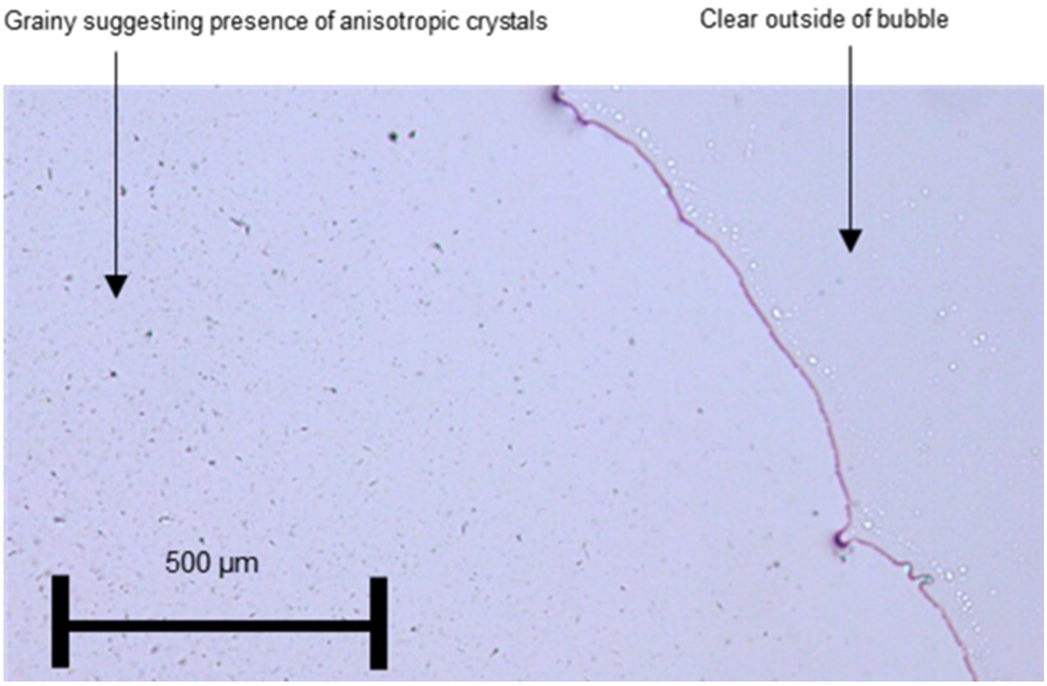

Fig. S5. Optical microscopy image highlighting the presence of a large amount of aggregates formed upon lyophilization and redispersion of tricaprin at 40 wt%, 50/50.

Table S7. Formulations containing dodecyl bis-prodrug (BPD) and tricaprin targeting at 33% composition in the LNP (before the addition of the sucrose). Sucrose was included as the cryoprotectant.

**Brij S20/ Lipoid S100**	**Ratio dodecyl bis-prodrug: tricaprin**	**Name**	**Sucrose conc. (% w/v)**	**total mass (mg)**	**Brij s20 (%)**	**Tricaparin (%)**	**prodrug (%)**	**Lipoid S100 (%)**	**Sucrose (%)**
50/50	1:1	50/50, 1:1	10	203	0.5	0.2	0.2	0.5	98.5
25/75	1:1	25/75, 1:1	10	203	0.2	0.2	0.2	0.7	98.5
50/50	3:1	50/50, 3:1	10	203	0.5	0.1	0.4	0.5	98.5
25/75	3:1	25/75, 3:1	10	203	0.2	0.1	0.4	0.7	98.5

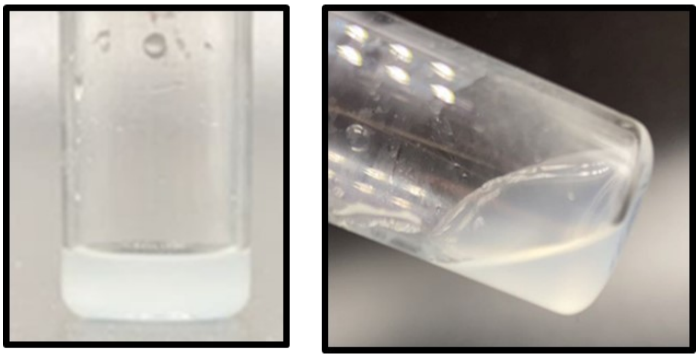

Figure S6. Photographs of core composition of 3:1 stabilised at 50/50 with 10 % w/v sucrose upon dispersion in 0.1 M PBS at 0.48 mg/mL lamivudine and 120 mg/ml in terms of the full formulation. Photos indicate no sign of aggregates.

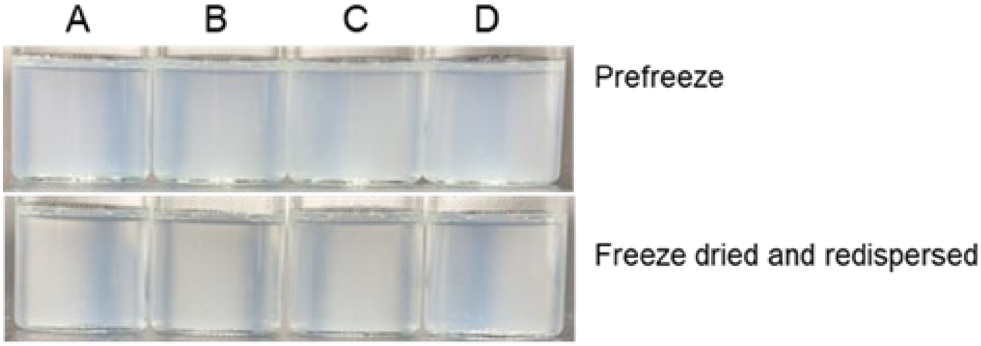

Fig. S7. Photographs of each dispersion containing various blends post redispersion. A) 50/50, 1:1. B) 25/75, 1:1. C) 50/50, 3:1. D) 25/75, 3:1.

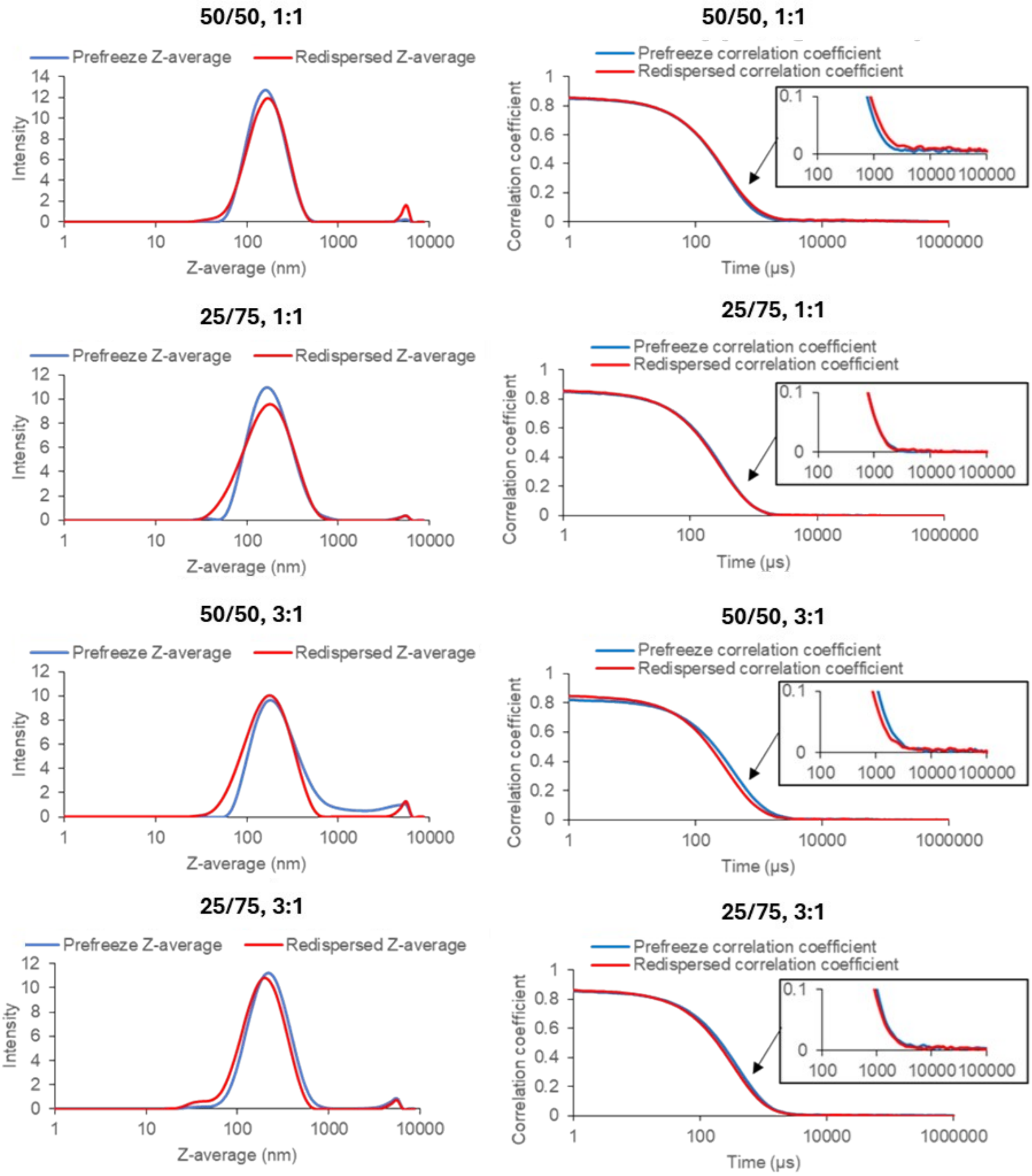

Fig. S8. Particle size distribution graphs and correlation coefficient graphs before and after freeze drying and redispersion for 33 wt % blends of dodecyl prodrug and tricaprin in the presence of 10 % w/v sucrose. Formulation with a core composition of 1:1 (BPD:tricaprin) stabilised by 50/50 or 25/75 or Formulation with a core composition of 3:1 stabilised at 50/50 or 25/75.

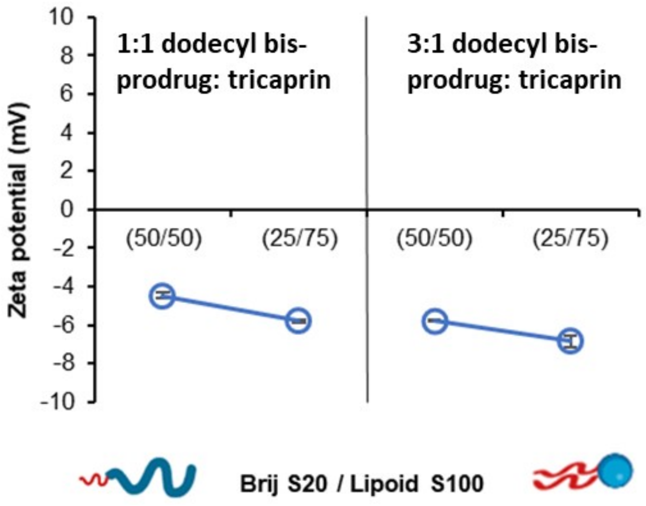

Fig S9. Zeta potential on formulations of dodecyl prodrug tricaprin blend formulations measured at 40 wt % for each surfactant composition of Brij S20 and Lipoid S100 (pre-freezing).

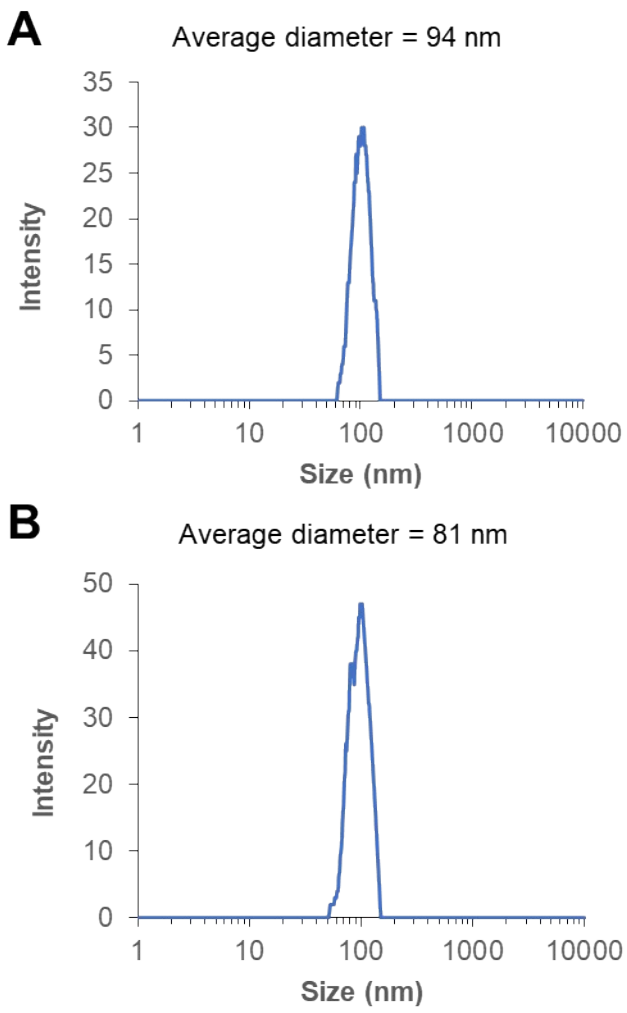

Fig. S10. Size distribution graphs using data calculated by ImageJ for the formulation with a core at 33 wt % and composition of 50/50, 1:1; A) Pre-freeze drying. B) Freeze dried and redispersed. Measured 100 nanoparticles per image.

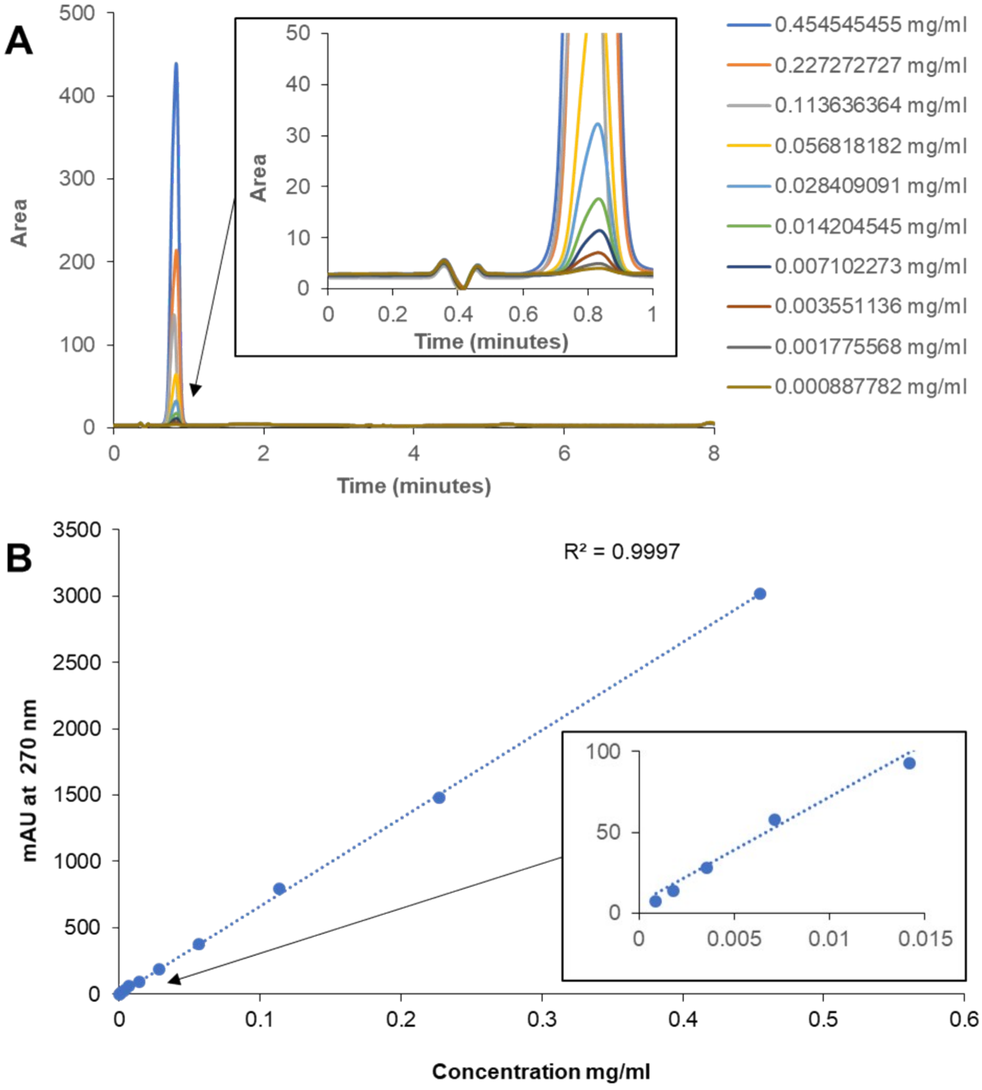

Fig. S11. HPLC analysis of lamivudine showing that concentration of lamivudine from 0.45455 mg/ml to 0.00089 mg/ml were within our calibration curve. A) Overlay of HPLC traces varying in lamivudine concentration. B) Calibration plot of UV signal vs concentration of lamivudine drug.

References

1 L. Shi, X. Wu, T. Li, Y. Wu, L. Song, W. Zhang, L. Yin, Y. Wu, W. Han and Y. Yang, *Nanoscale Adv.*, **4**, 952–966.

2 L. Zhang, L. Zhu, L. Tang, J. Xie, Y. Gao, C. Yu, K. Shang, H. Han, C. Liu and Y. Lu, *Adv. Sci.*, 2023, **10**, 2205246.
